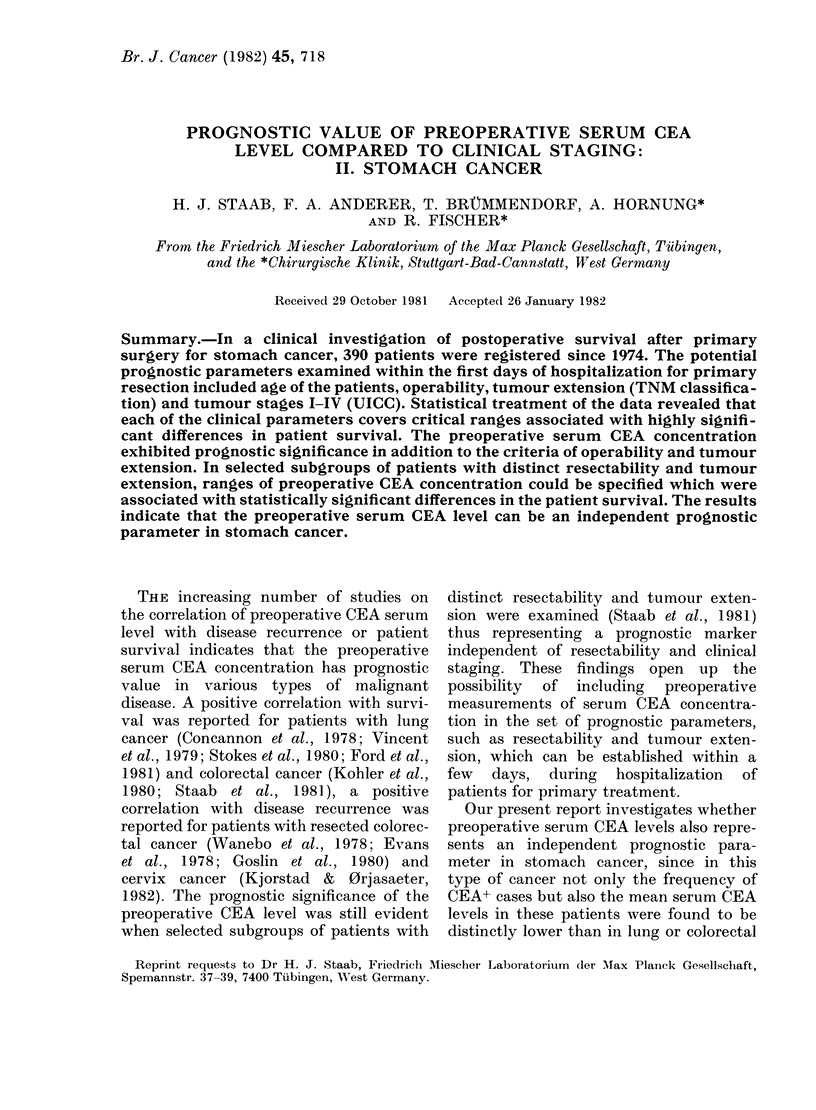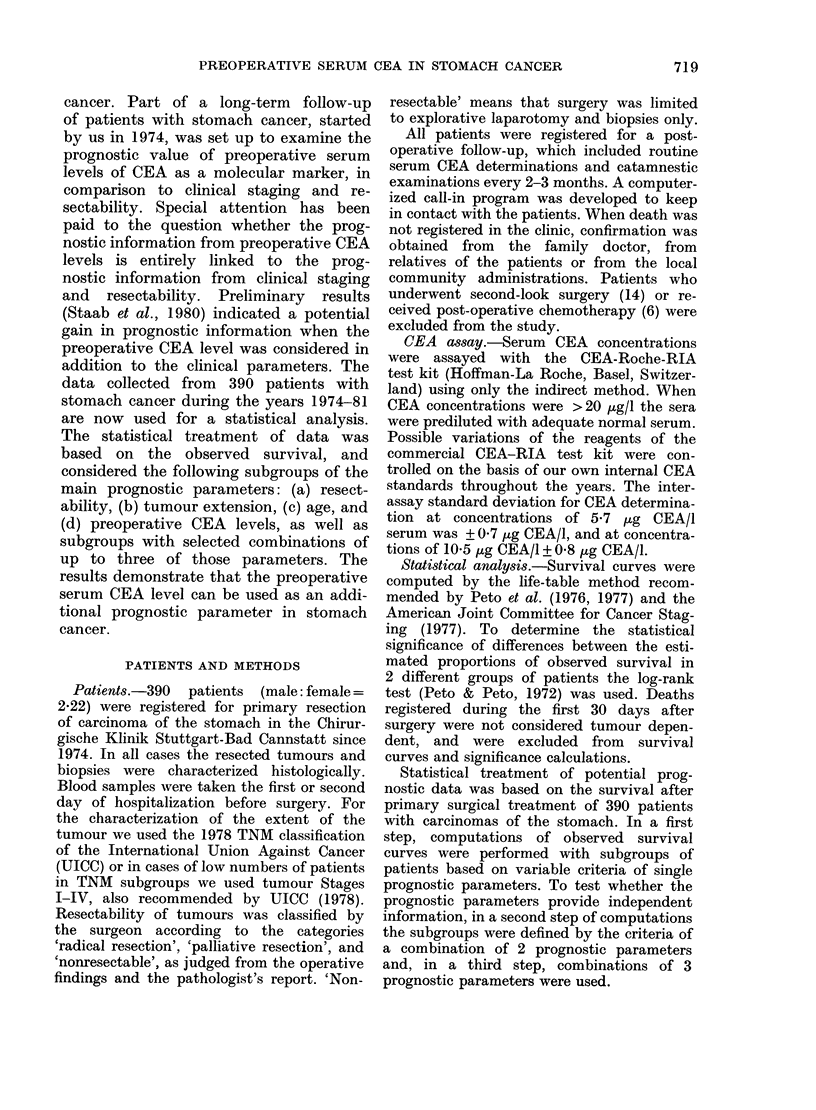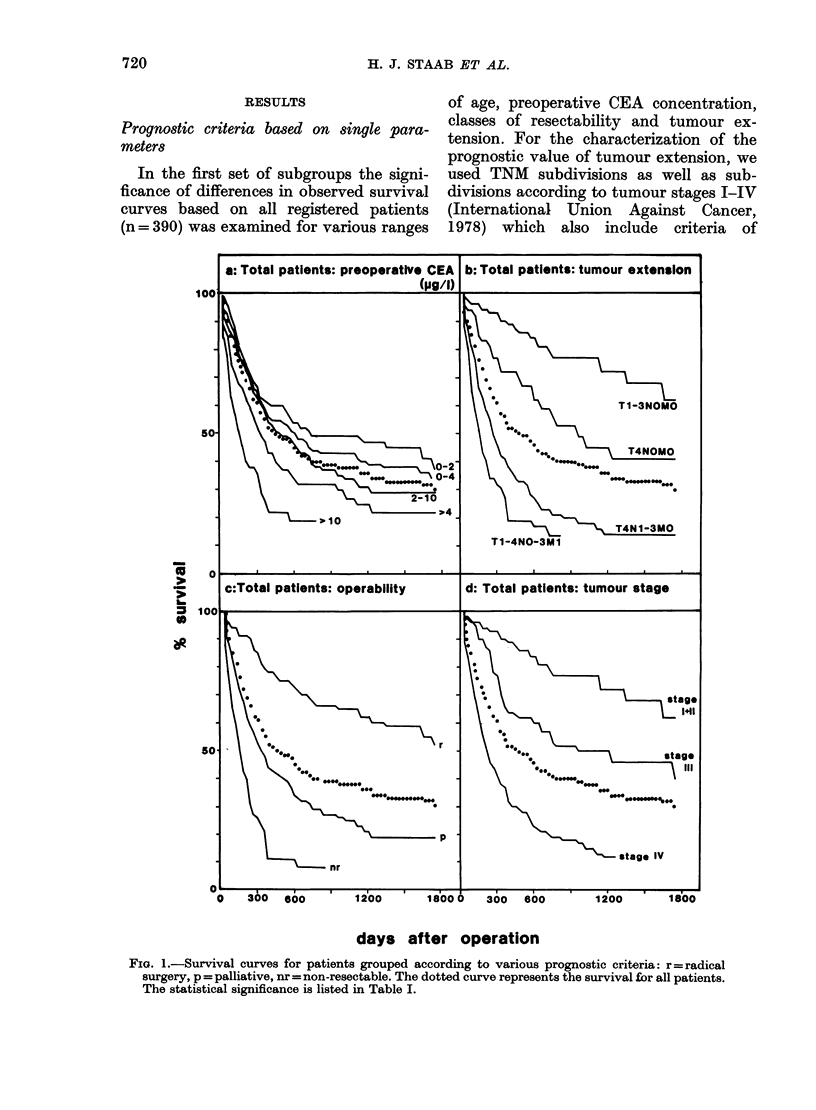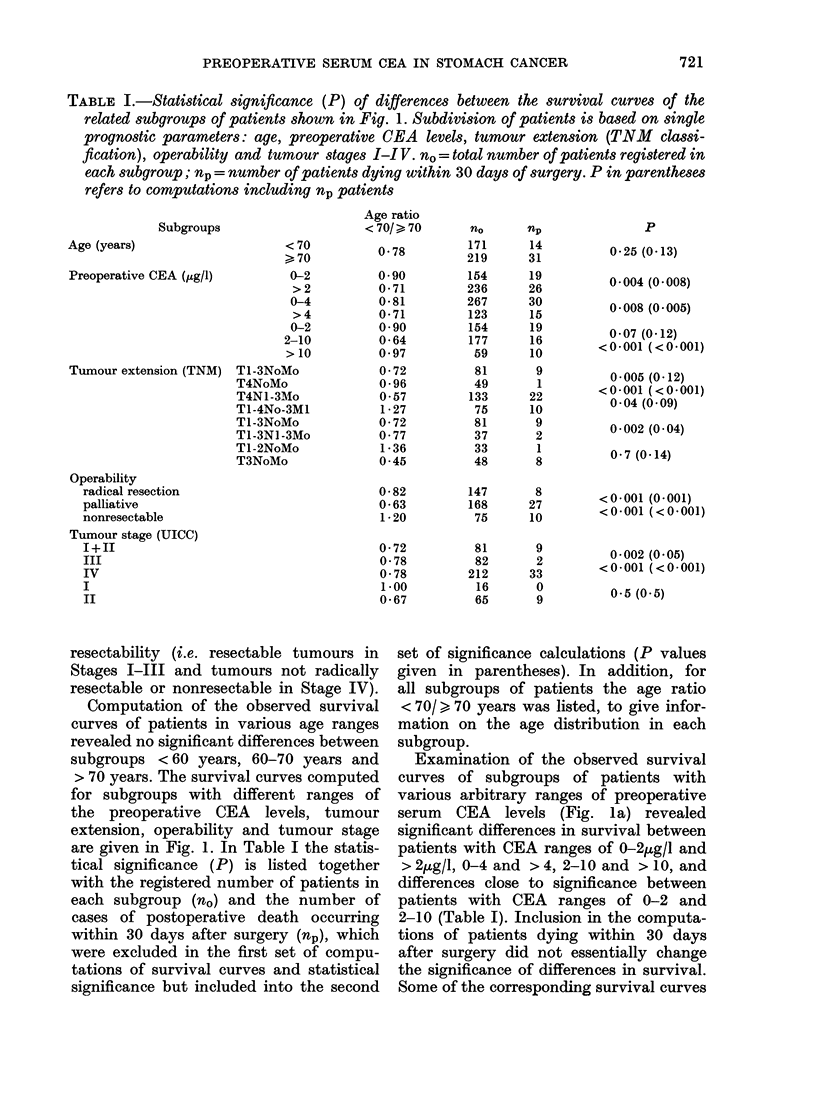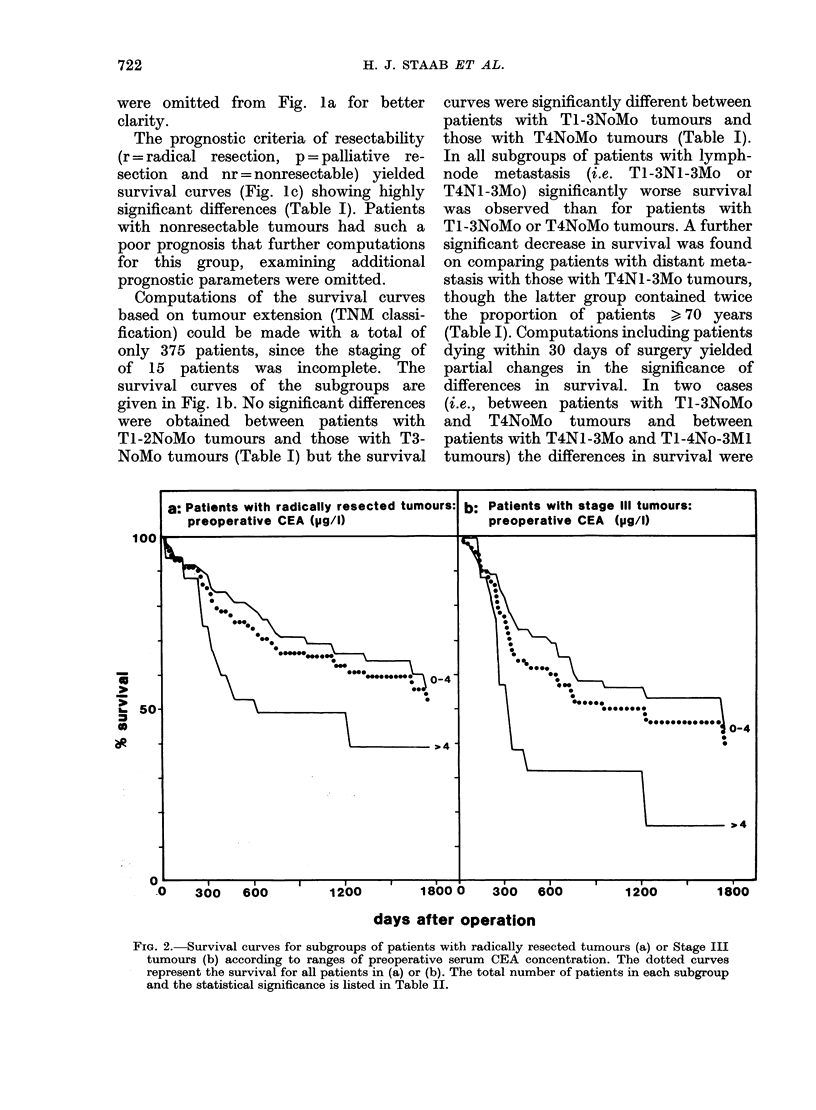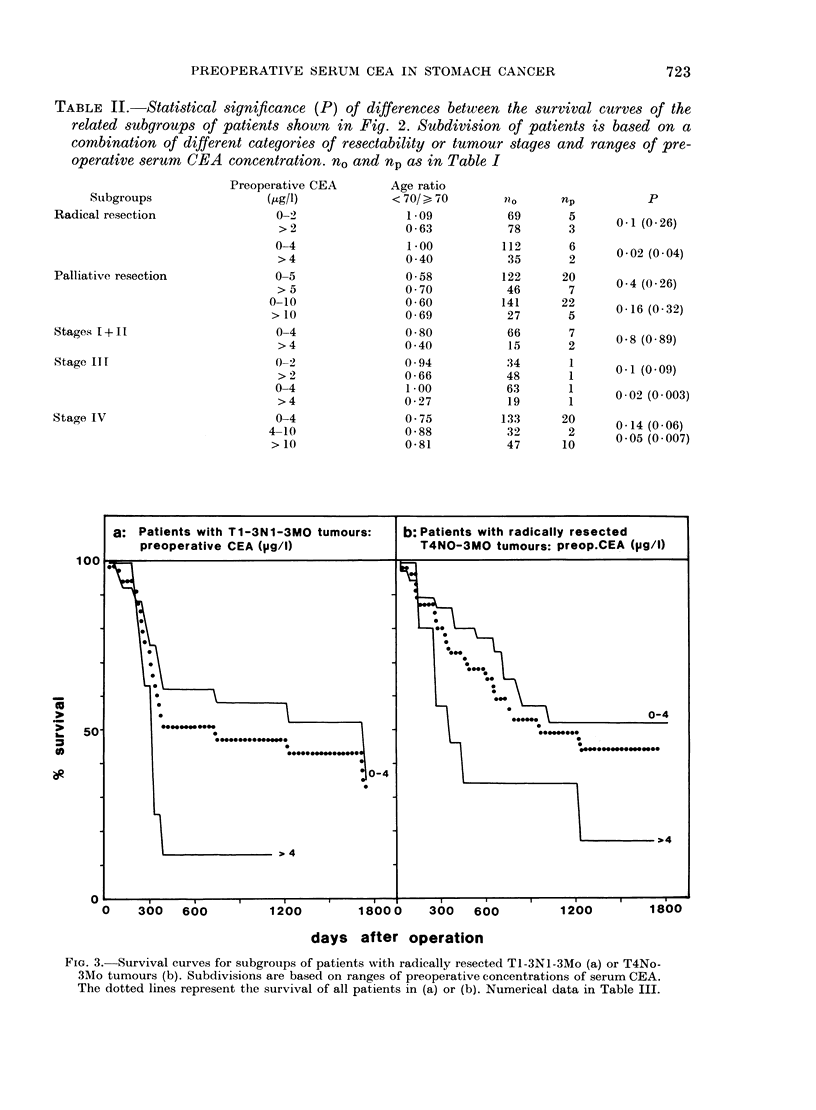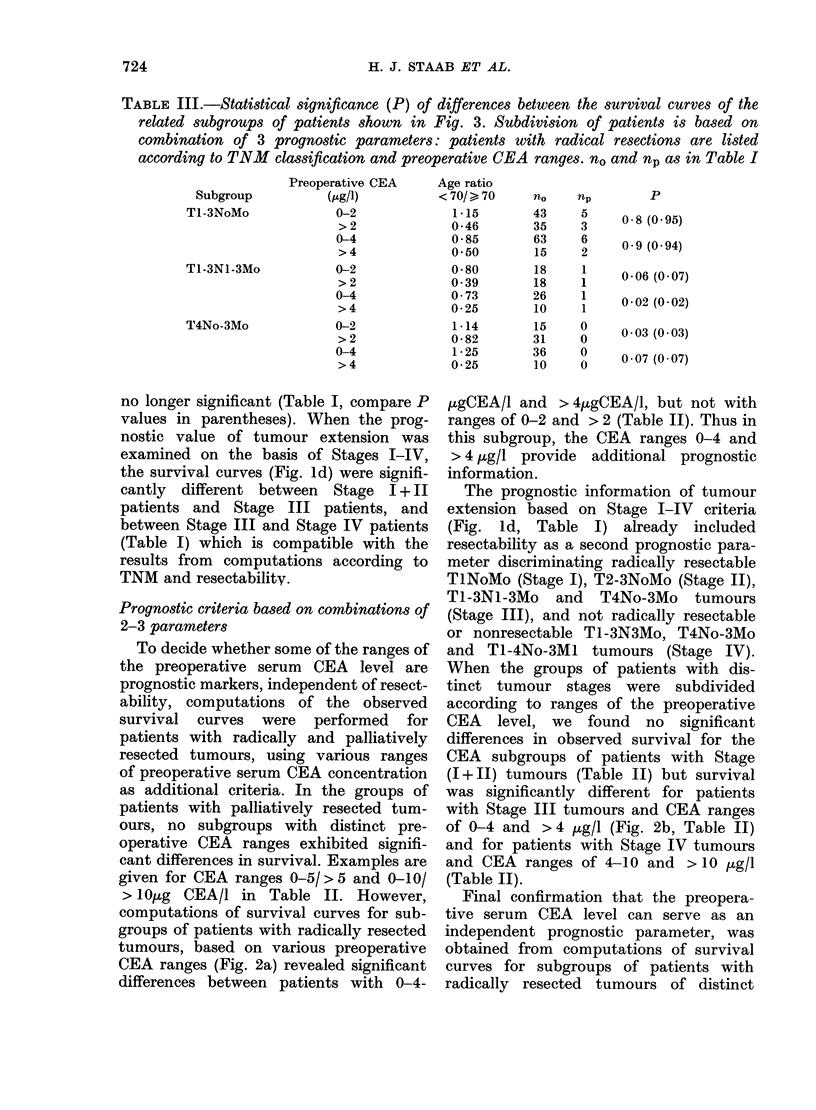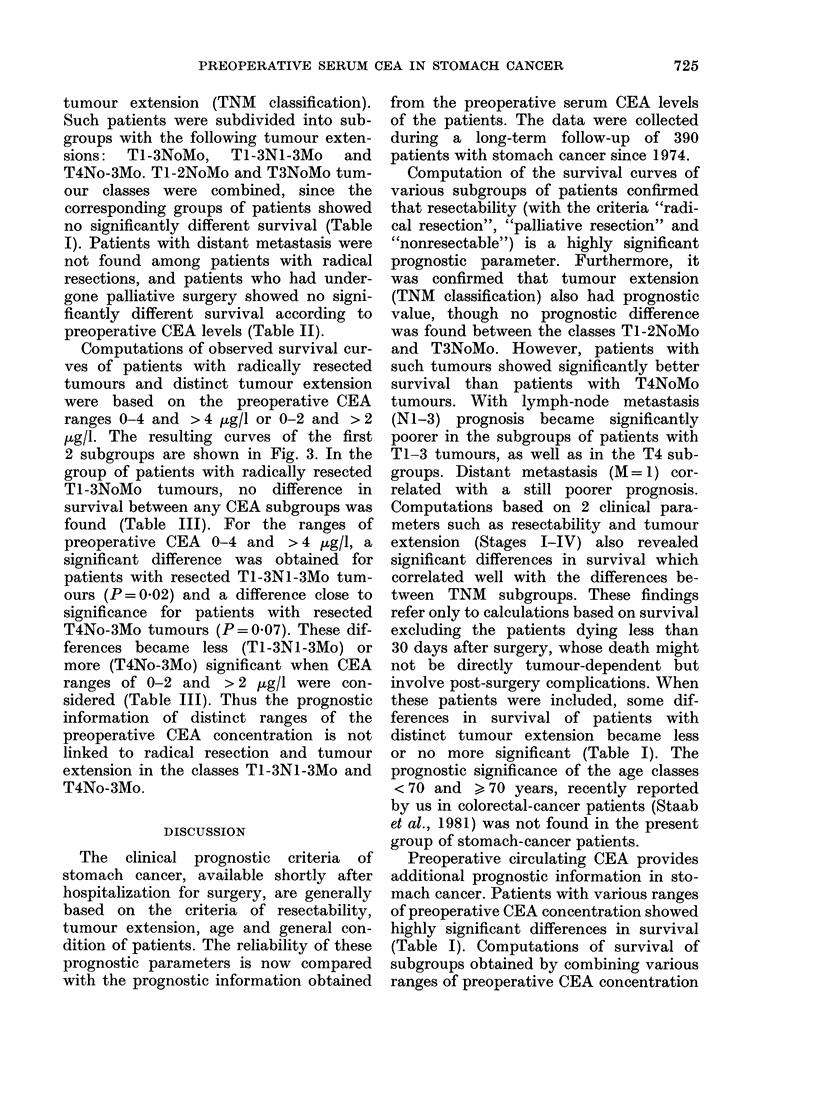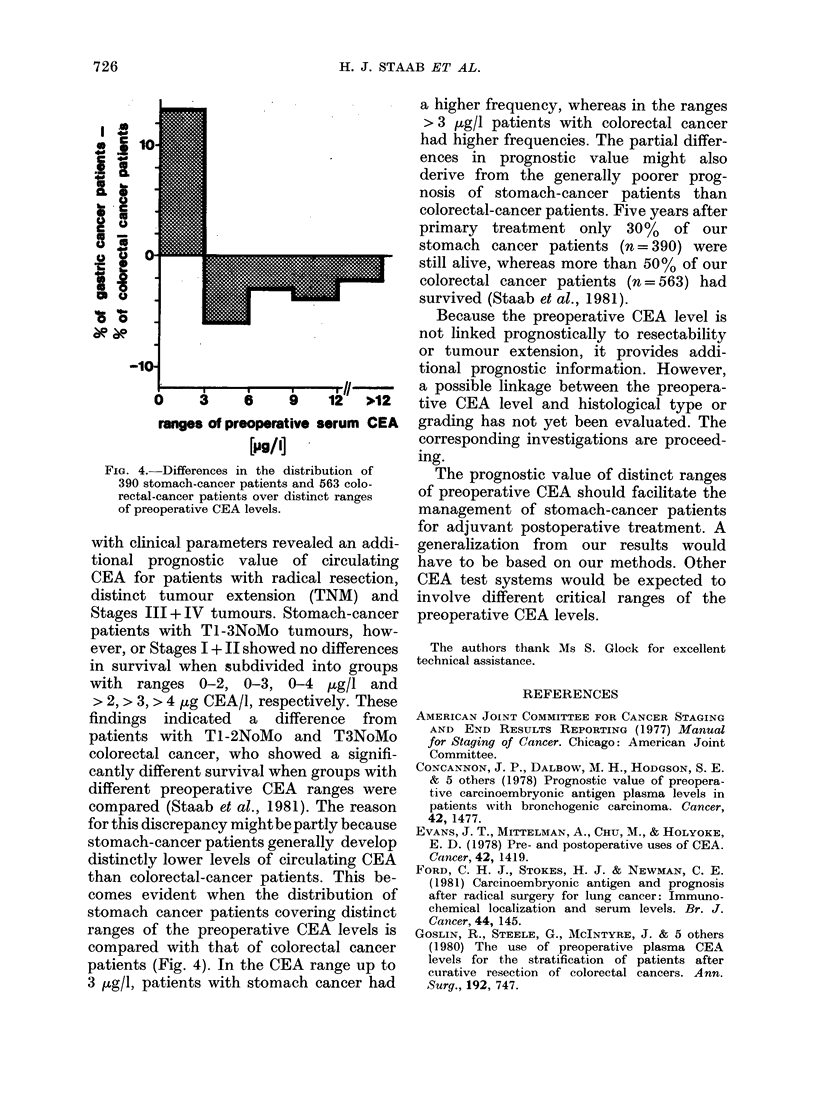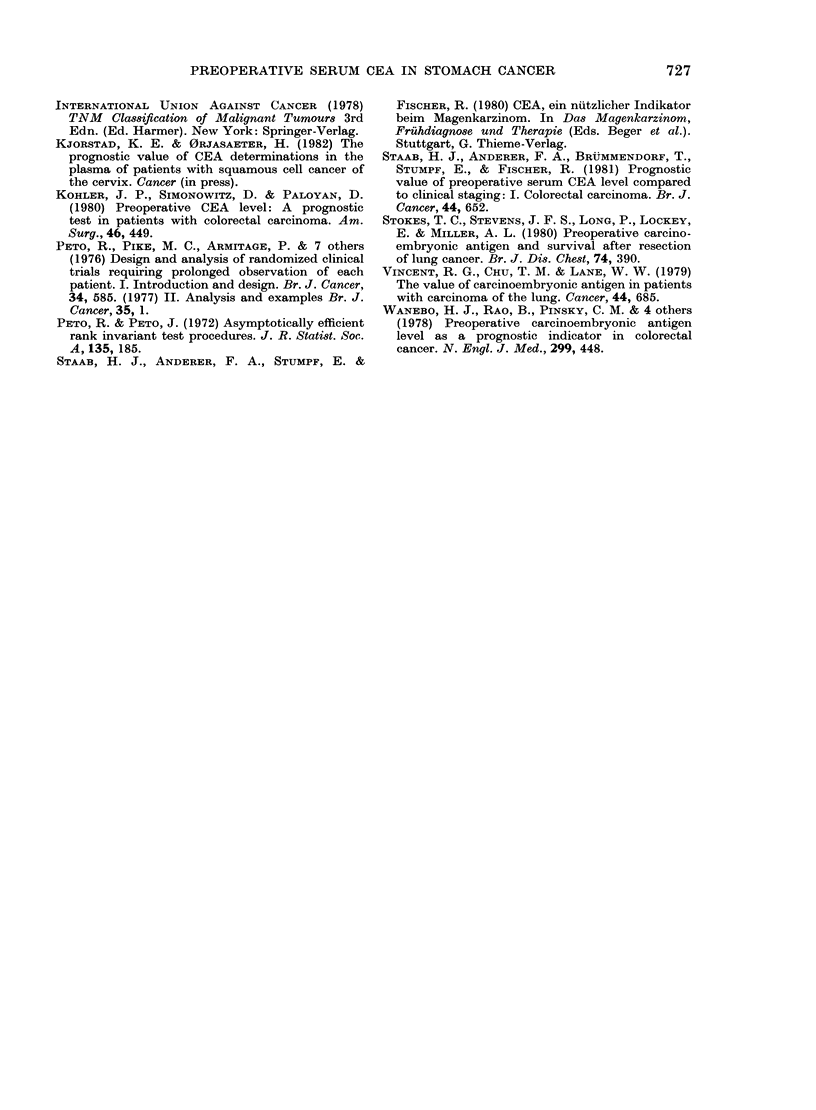# Prognostic value of preoperative serum CEA level compared to clinical staging: II. Stomach cancer.

**DOI:** 10.1038/bjc.1982.113

**Published:** 1982-05

**Authors:** H. J. Staab, F. A. Anderer, T. Brümmendorf, A. Hornung, R. Fischer

## Abstract

In a clinical investigation of postoperative survival after primary surgery for stomach cancer, 390 patients were registered since 1974. The potential prognostic parameters examined within the first days of hospitalization for primary resection included age of the patients, operability, tumour extension (TNM classification) and tumour stages I-IV (UICC). Statistical treatment of the data revealed that each of the clinical parameters covers critical ranges associated with highly significant differences in patient survival. The preoperative serum CEA concentration exhibited prognostic significance in addition to the criteria of operability and tumour extension. In selected subgroups of patients with distinct resectability and tumour extension, ranges of preoperative CEA concentration could be specified which were associated with statistically significant differences in the patient survival. The results indicate that the preoperative serum CEA level can be an independent prognostic parameter in stomach cancer.


*Br. J. Cancer* (1982) **45**, 718

### PROGNOSTIC VALUE OF PREOPERATIVE SERUM CEA LEVEL COMPARED TO CLINICAL STAGING: II. STOMACH CANCER

H. J. STAAB, F. A. ANDERER, T. BRÜMMENDORF, A. HORNUNG* AND R. FISCHER*

*From the Friedrich Miescher Laboratorium of the Max Planck Gesellschaft, Tübingen, and the *Chirurgische Klinik, Stuttgart-Bad-Cannstatt, West Germany*

Received 29 October 1981 Accepted 26 January 1982

**Summary.—In a clinical investigation of postoperative survival after primary surgery for stomach cancer, 390 patients were registered since 1974. The potential prognostic parameters examined within the first days of hospitalization for primary resection included age of the patients, operability, tumour extension (TNM classification) and tumour stages I–IV (UICC). Statistical treatment of the data revealed that each of the clinical parameters covers critical ranges associated with highly significant differences in patient survival. The preoperative serum CEA concentration exhibited prognostic significance in addition to the criteria of operability and tumour extension. In selected subgroups of patients with distinct resectability and tumour extension, ranges of preoperative CEA concentration could be specified which were associated with statistically significant differences in the patient survival. The results indicate that the preoperative serum CEA level can be an independent prognostic parameter in stomach cancer.**

THE increasing number of studies on the correlation of preoperative CEA serum level with disease recurrence or patient survival indicates that the preoperative serum CEA concentration has prognostic value in various types of malignant disease. A positive correlation with survival was reported for patients with lung cancer (Concannon *et al.*, 1978; Vincent *et al.*, 1979; Stokes *et al.*, 1980; Ford *et al.*, 1981) and colorectal cancer (Kohler *et al.*, 1980; Staab *et al.*, 1981), a positive correlation with disease recurrence was reported for patients with resected colorectal cancer (Wanebo *et al.*, 1978; Evans *et al.*, 1978; Goslin *et al.*, 1980) and cervix cancer (Kjorstad & Ørjasaeter, 1982). The prognostic significance of the preoperative CEA level was still evident when selected subgroups of patients with distinct resectability and tumour extension were examined (Staab *et al.*, 1981) thus representing a prognostic marker independent of resectability and clinical staging. These findings open up the possibility of including preoperative measurements of serum CEA concentration in the set of prognostic parameters, such as resectability and tumour extension, which can be established within a few days, during hospitalization of patients for primary treatment.

Our present report investigates whether preoperative serum CEA levels also represents an independent prognostic parameter in stomach cancer, since in this type of cancer not only the frequency of $CEA^+$ cases but also the mean serum CEA levels in these patients were found to be distinctly lower than in lung or colorectal

Reprint requests to Dr H. J. Staab, Friedrich Miescher Laboratorium der Max Planck Gesellschaft, Spemannstr. 37–39, 7400 Tübingen, West Germany.

cancer. Part of a long-term follow-up of patients with stomach cancer, started by us in 1974, was set up to examine the prognostic value of preoperative serum levels of CEA as a molecular marker, in comparison to clinical staging and resectability. Special attention has been paid to the question whether the prognostic information from preoperative CEA levels is entirely linked to the prognostic information from clinical staging and resectability. Preliminary results (Staab *et al.*, 1980) indicated a potential gain in prognostic information when the preoperative CEA level was considered in addition to the clinical parameters. The data collected from 390 patients with stomach cancer during the years 1974–81 are now used for a statistical analysis. The statistical treatment of data was based on the observed survival, and considered the following subgroups of the main prognostic parameters: (a) resectability, (b) tumour extension, (c) age, and (d) preoperative CEA levels, as well as subgroups with selected combinations of up to three of those parameters. The results demonstrate that the preoperative serum CEA level can be used as an additional prognostic parameter in stomach cancer.

### PATIENTS AND METHODS

*Patients.*—390 patients (male:female = 2·22) were registered for primary resection of carcinoma of the stomach in the Chirurgische Klinik Stuttgart-Bad Cannstatt since 1974. In all cases the resected tumours and biopsies were characterized histologically. Blood samples were taken the first or second day of hospitalization before surgery. For the characterization of the extent of the tumour we used the 1978 TNM classification of the International Union Against Cancer (UICC) or in cases of low numbers of patients in TNM subgroups we used tumour Stages I–IV, also recommended by UICC (1978). Resectability of tumours was classified by the surgeon according to the categories 'radical resection', 'palliative resection', and 'nonresectable', as judged from the operative findings and the pathologist's report. 'Nonresectable' means that surgery was limited to explorative laparotomy and biopsies only.

All patients were registered for a postoperative follow-up, which included routine serum CEA determinations and catamnestic examinations every 2–3 months. A computerized call-in program was developed to keep in contact with the patients. When death was not registered in the clinic, confirmation was obtained from the family doctor, from relatives of the patients or from the local community administrations. Patients who underwent second-look surgery (14) or received post-operative chemotherapy (6) were excluded from the study.

*CEA assay.*—Serum CEA concentrations were assayed with the CEA-Roche-RIA test kit (Hoffman-La Roche, Basel, Switzerland) using only the indirect method. When CEA concentrations were $>20$ μg/l the sera were prediluted with adequate normal serum. Possible variations of the reagents of the commercial CEA–RIA test kit were controlled on the basis of our own internal CEA standards throughout the years. The interassay standard deviation for CEA determination at concentrations of 5·7 μg CEA/l serum was $\pm 0{\cdot}7$ μg CEA/l, and at concentrations of 10·5 μg CEA/l $\pm 0{\cdot}8$ μg CEA/l.

*Statistical analysis.*—Survival curves were computed by the life-table method recommended by Peto *et al.* (1976, 1977) and the American Joint Committee for Cancer Staging (1977). To determine the statistical significance of differences between the estimated proportions of observed survival in 2 different groups of patients the log-rank test (Peto & Peto, 1972) was used. Deaths registered during the first 30 days after surgery were not considered tumour dependent, and were excluded from survival curves and significance calculations.

Statistical treatment of potential prognostic data was based on the survival after primary surgical treatment of 390 patients with carcinomas of the stomach. In a first step, computations of observed survival curves were performed with subgroups of patients based on variable criteria of single prognostic parameters. To test whether the prognostic parameters provide independent information, in a second step of computations the subgroups were defined by the criteria of a combination of 2 prognostic parameters and, in a third step, combinations of 3 prognostic parameters were used.

### RESULTS

*Prognostic criteria based on single parameters*

In the first set of subgroups the significance of differences in observed survival curves based on all registered patients (n = 390) was examined for various ranges of age, preoperative CEA concentration, classes of resectability and tumour extension. For the characterization of the prognostic value of tumour extension, we used TNM subdivisions as well as subdivisions according to tumour stages I–IV (International Union Against Cancer, 1978) which also include criteria of

Fig. 1.—Survival curves for patients grouped according to various prognostic criteria: r = radical surgery, p = palliative, nr = non-resectable. The dotted curve represents the survival for all patients. The statistical significance is listed in Table I.

TABLE I.—*Statistical significance (P) of differences between the survival curves of the related subgroups of patients shown in Fig.* 1. *Subdivision of patients is based on single prognostic parameters: age, preoperative CEA levels, tumour extension (TNM classification), operability and tumour stages I–IV.* $n_o$ = *total number of patients registered in each subgroup;* $n_p$ = *number of patients dying within* 30 *days of surgery. P in parentheses refers to computations including* $n_p$ *patients*

| Subgroups | | Age ratio < 70/≥ 70 | $n_o$ | $n_p$ | *P* |
|---|---|---|---|---|---|
| Age (years) | < 70 | 0·78 | 171 | 14 | 0·25 (0·13) |
| | ≥ 70 | | 219 | 31 | |
| Preoperative CEA (μg/l) | 0–2 | 0·90 | 154 | 19 | 0·004 (0·008) |
| | > 2 | 0·71 | 236 | 26 | |
| | 0–4 | 0·81 | 267 | 30 | 0·008 (0·005) |
| | > 4 | 0·71 | 123 | 15 | |
| | 0–2 | 0·90 | 154 | 19 | 0·07 (0·12) |
| | 2–10 | 0·64 | 177 | 16 | < 0·001 (< 0·001) |
| | > 10 | 0·97 | 59 | 10 | |
| Tumour extension (TNM) | T1-3NoMo | 0·72 | 81 | 9 | 0·005 (0·12) |
| | T4NoMo | 0·96 | 49 | 1 | < 0·001 (< 0·001) |
| | T4N1-3Mo | 0·57 | 133 | 22 | 0·04 (0·09) |
| | T1-4No-3M1 | 1·27 | 75 | 10 | |
| | T1-3NoMo | 0·72 | 81 | 9 | 0·002 (0·04) |
| | T1-3N1-3Mo | 0·77 | 37 | 2 | |
| | T1-2NoMo | 1·36 | 33 | 1 | 0·7 (0·14) |
| | T3NoMo | 0·45 | 48 | 8 | |
| Operability | | | | | |
| radical resection | | 0·82 | 147 | 8 | < 0·001 (0·001) |
| palliative | | 0·63 | 168 | 27 | < 0·001 (< 0·001) |
| nonresectable | | 1·20 | 75 | 10 | |
| Tumour stage (UICC) | | | | | |
| I+II | | 0·72 | 81 | 9 | 0·002 (0·05) |
| III | | 0·78 | 82 | 2 | < 0·001 (< 0·001) |
| IV | | 0·78 | 212 | 33 | |
| I | | 1·00 | 16 | 0 | 0·5 (0·5) |
| II | | 0·67 | 65 | 9 | |

resectability (*i.e.* resectable tumours in Stages I–III and tumours not radically resectable or nonresectable in Stage IV).

Computation of the observed survival curves of patients in various age ranges revealed no significant differences between subgroups < 60 years, 60–70 years and > 70 years. The survival curves computed for subgroups with different ranges of the preoperative CEA levels, tumour extension, operability and tumour stage are given in Fig. 1. In Table I the statistical significance (*P*) is listed together with the registered number of patients in each subgroup ($n_o$) and the number of cases of postoperative death occurring within 30 days after surgery ($n_p$), which were excluded in the first set of computations of survival curves and statistical significance but included into the second set of significance calculations (*P* values given in parentheses). In addition, for all subgroups of patients the age ratio < 70/ ≥ 70 years was listed, to give information on the age distribution in each subgroup.

Examination of the observed survival curves of subgroups of patients with various arbitrary ranges of preoperative serum CEA levels (Fig. 1a) revealed significant differences in survival between patients with CEA ranges of 0–2μg/l and > 2μg/l, 0–4 and > 4, 2–10 and > 10, and differences close to significance between patients with CEA ranges of 0–2 and 2–10 (Table I). Inclusion in the computations of patients dying within 30 days after surgery did not essentially change the significance of differences in survival. Some of the corresponding survival curves were omitted from Fig. 1a for better clarity.

The prognostic criteria of resectability (r = radical resection, p = palliative resection and nr = nonresectable) yielded survival curves (Fig. 1c) showing highly significant differences (Table I). Patients with nonresectable tumours had such a poor prognosis that further computations for this group, examining additional prognostic parameters were omitted.

Computations of the survival curves based on tumour extension (TNM classification) could be made with a total of only 375 patients, since the staging of of 15 patients was incomplete. The survival curves of the subgroups are given in Fig. 1b. No significant differences were obtained between patients with T1-2NoMo tumours and those with T3-NoMo tumours (Table I) but the survival curves were significantly different between patients with T1-3NoMo tumours and those with T4NoMo tumours (Table I). In all subgroups of patients with lymph-node metastasis (*i.e.* T1-3N1-3Mo or T4N1-3Mo) significantly worse survival was observed than for patients with T1-3NoMo or T4NoMo tumours. A further significant decrease in survival was found on comparing patients with distant metastasis with those with T4N1-3Mo tumours, though the latter group contained twice the proportion of patients ≥ 70 years (Table I). Computations including patients dying within 30 days of surgery yielded partial changes in the significance of differences in survival. In two cases (*i.e.*, between patients with T1-3NoMo and T4NoMo tumours and between patients with T4N1-3Mo and T1-4No-3M1 tumours) the differences in survival were

FIG. 2.—Survival curves for subgroups of patients with radically resected tumours (a) or Stage III tumours (b) according to ranges of preoperative serum CEA concentration. The dotted curves represent the survival for all patients in (a) or (b). The total number of patients in each subgroup and the statistical significance is listed in Table II.

TABLE II.—*Statistical significance (P) of differences between the survival curves of the related subgroups of patients shown in Fig. 2. Subdivision of patients is based on a combination of different categories of resectability or tumour stages and ranges of preoperative serum CEA concentration.* $n_o$ *and* $n_p$ *as in Table I*

| Subgroups | Preoperative CEA (μg/l) | Age ratio <70/≥70 | $n_o$ | $n_p$ | P |
|---|---|---|---|---|---|
| Radical resection | 0–2 | 1·09 | 69 | 5 | 0·1 (0·26) |
| | >2 | 0·63 | 78 | 3 | |
| | 0–4 | 1·00 | 112 | 6 | 0·02 (0·04) |
| | >4 | 0·40 | 35 | 2 | |
| Palliative resection | 0–5 | 0·58 | 122 | 20 | 0·4 (0·26) |
| | >5 | 0·70 | 46 | 7 | |
| | 0–10 | 0·60 | 141 | 22 | 0·16 (0·32) |
| | >10 | 0·69 | 27 | 5 | |
| Stages I+II | 0–4 | 0·80 | 66 | 7 | 0·8 (0·89) |
| | >4 | 0·40 | 15 | 2 | |
| Stage III | 0–2 | 0·94 | 34 | 1 | 0·1 (0·09) |
| | >2 | 0·66 | 48 | 1 | |
| | 0–4 | 1·00 | 63 | 1 | 0·02 (0·003) |
| | >4 | 0·27 | 19 | 1 | |
| Stage IV | 0–4 | 0·75 | 133 | 20 | 0·14 (0·06) |
| | 4–10 | 0·88 | 32 | 2 | 0·05 (0·007) |
| | >10 | 0·81 | 47 | 10 | |

FIG. 3.—Survival curves for subgroups of patients with radically resected T1-3N1-3Mo (a) or T4No-3Mo tumours (b). Subdivisions are based on ranges of preoperative concentrations of serum CEA. The dotted lines represent the survival of all patients in (a) or (b). Numerical data in Table III.

TABLE III.—*Statistical significance (P) of differences between the survival curves of the related subgroups of patients shown in Fig. 3. Subdivision of patients is based on combination of 3 prognostic parameters: patients with radical resections are listed according to TNM classification and preoperative CEA ranges.* $n_o$ *and* $n_p$ *as in Table I*

| Subgroup | Preoperative CEA (μg/l) | Age ratio <70/≥70 | $n_o$ | $n_p$ | P |
|---|---|---|---|---|---|
| T1-3NoMo | 0–2 | 1·15 | 43 | 5 | 0·8 (0·95) |
| | >2 | 0·46 | 35 | 3 | |
| | 0–4 | 0·85 | 63 | 6 | 0·9 (0·94) |
| | >4 | 0·50 | 15 | 2 | |
| T1-3N1-3Mo | 0–2 | 0·80 | 18 | 1 | 0·06 (0·07) |
| | >2 | 0·39 | 18 | 1 | |
| | 0–4 | 0·73 | 26 | 1 | 0·02 (0·02) |
| | >4 | 0·25 | 10 | 1 | |
| T4No-3Mo | 0–2 | 1·14 | 15 | 0 | 0·03 (0·03) |
| | >2 | 0·82 | 31 | 0 | |
| | 0–4 | 1·25 | 36 | 0 | 0·07 (0·07) |
| | >4 | 0·25 | 10 | 0 | |

no longer significant (Table I, compare *P* values in parentheses). When the prognostic value of tumour extension was examined on the basis of Stages I–IV, the survival curves (Fig. 1d) were significantly different between Stage I+II patients and Stage III patients, and between Stage III and Stage IV patients (Table I) which is compatible with the results from computations according to TNM and resectability.

*Prognostic criteria based on combinations of 2–3 parameters*

To decide whether some of the ranges of the preoperative serum CEA level are prognostic markers, independent of resectability, computations of the observed survival curves were performed for patients with radically and palliatively resected tumours, using various ranges of preoperative serum CEA concentration as additional criteria. In the groups of patients with palliatively resected tumours, no subgroups with distinct preoperative CEA ranges exhibited significant differences in survival. Examples are given for CEA ranges 0–5/>5 and 0–10/>10μg CEA/l in Table II. However, computations of survival curves for subgroups of patients with radically resected tumours, based on various preoperative CEA ranges (Fig. 2a) revealed significant differences between patients with 0–4-μgCEA/l and >4μgCEA/l, but not with ranges of 0–2 and >2 (Table II). Thus in this subgroup, the CEA ranges 0–4 and >4 μg/l provide additional prognostic information.

The prognostic information of tumour extension based on Stage I–IV criteria (Fig. 1d, Table I) already included resectability as a second prognostic parameter discriminating radically resectable T1NoMo (Stage I), T2-3NoMo (Stage II), T1-3N1-3Mo and T4No-3Mo tumours (Stage III), and not radically resectable or nonresectable T1-3N3Mo, T4No-3Mo and T1-4No-3M1 tumours (Stage IV). When the groups of patients with distinct tumour stages were subdivided according to ranges of the preoperative CEA level, we found no significant differences in observed survival for the CEA subgroups of patients with Stage (I+II) tumours (Table II) but survival was significantly different for patients with Stage III tumours and CEA ranges of 0–4 and >4 μg/l (Fig. 2b, Table II) and for patients with Stage IV tumours and CEA ranges of 4–10 and >10 μg/l (Table II).

Final confirmation that the preoperative serum CEA level can serve as an independent prognostic parameter, was obtained from computations of survival curves for subgroups of patients with radically resected tumours of distinct tumour extension (TNM classification). Such patients were subdivided into subgroups with the following tumour extensions: T1-3NoMo, T1-3N1-3Mo and T4No-3Mo. T1-2NoMo and T3NoMo tumour classes were combined, since the corresponding groups of patients showed no significantly different survival (Table I). Patients with distant metastasis were not found among patients with radical resections, and patients who had undergone palliative surgery showed no significantly different survival according to preoperative CEA levels (Table II).

Computations of observed survival curves of patients with radically resected tumours and distinct tumour extension were based on the preoperative CEA ranges 0–4 and $>4$ $\mu$g/l or 0–2 and $>2$ $\mu$g/l. The resulting curves of the first 2 subgroups are shown in Fig. 3. In the group of patients with radically resected T1-3NoMo tumours, no difference in survival between any CEA subgroups was found (Table III). For the ranges of preoperative CEA 0–4 and $>4$ $\mu$g/l, a significant difference was obtained for patients with resected T1-3N1-3Mo tumours ($P=0{\cdot}02$) and a difference close to significance for patients with resected T4No-3Mo tumours ($P=0{\cdot}07$). These differences became less (T1-3N1-3Mo) or more (T4No-3Mo) significant when CEA ranges of 0–2 and $>2$ $\mu$g/l were considered (Table III). Thus the prognostic information of distinct ranges of the preoperative CEA concentration is not linked to radical resection and tumour extension in the classes T1-3N1-3Mo and T4No-3Mo.

### DISCUSSION

The clinical prognostic criteria of stomach cancer, available shortly after hospitalization for surgery, are generally based on the criteria of resectability, tumour extension, age and general condition of patients. The reliability of these prognostic parameters is now compared with the prognostic information obtained from the preoperative serum CEA levels of the patients. The data were collected during a long-term follow-up of 390 patients with stomach cancer since 1974.

Computation of the survival curves of various subgroups of patients confirmed that resectability (with the criteria "radical resection", "palliative resection" and "nonresectable") is a highly significant prognostic parameter. Furthermore, it was confirmed that tumour extension (TNM classification) also had prognostic value, though no prognostic difference was found between the classes T1-2NoMo and T3NoMo. However, patients with such tumours showed significantly better survival than patients with T4NoMo tumours. With lymph-node metastasis (N1–3) prognosis became significantly poorer in the subgroups of patients with T1–3 tumours, as well as in the T4 subgroups. Distant metastasis ($M=1$) correlated with a still poorer prognosis. Computations based on 2 clinical parameters such as resectability and tumour extension (Stages I–IV) also revealed significant differences in survival which correlated well with the differences between TNM subgroups. These findings refer only to calculations based on survival excluding the patients dying less than 30 days after surgery, whose death might not be directly tumour-dependent but involve post-surgery complications. When these patients were included, some differences in survival of patients with distinct tumour extension became less or no more significant (Table I). The prognostic significance of the age classes $<70$ and $\geqslant 70$ years, recently reported by us in colorectal-cancer patients (Staab *et al.*, 1981) was not found in the present group of stomach-cancer patients.

Preoperative circulating CEA provides additional prognostic information in stomach cancer. Patients with various ranges of preoperative CEA concentration showed highly significant differences in survival (Table I). Computations of survival of subgroups obtained by combining various ranges of preoperative CEA concentration

FIG. 4.—Differences in the distribution of 390 stomach-cancer patients and 563 colorectal-cancer patients over distinct ranges of preoperative CEA levels.

with clinical parameters revealed an additional prognostic value of circulating CEA for patients with radical resection, distinct tumour extension (TNM) and Stages III + IV tumours. Stomach-cancer patients with T1-3NoMo tumours, however, or Stages I + II showed no differences in survival when subdivided into groups with ranges 0–2, 0–3, 0–4 $\mu$g/l and > 2, > 3, > 4 $\mu$g CEA/l, respectively. These findings indicated a difference from patients with T1-2NoMo and T3NoMo colorectal cancer, who showed a significantly different survival when groups with different preoperative CEA ranges were compared (Staab *et al.*, 1981). The reason for this discrepancy might be partly because stomach-cancer patients generally develop distinctly lower levels of circulating CEA than colorectal-cancer patients. This becomes evident when the distribution of stomach cancer patients covering distinct ranges of the preoperative CEA levels is compared with that of colorectal cancer patients (Fig. 4). In the CEA range up to 3 $\mu$g/l, patients with stomach cancer had a higher frequency, whereas in the ranges > 3 $\mu$g/l patients with colorectal cancer had higher frequencies. The partial differences in prognostic value might also derive from the generally poorer prognosis of stomach-cancer patients than colorectal-cancer patients. Five years after primary treatment only 30% of our stomach cancer patients ($n = 390$) were still alive, whereas more than 50% of our colorectal cancer patients ($n = 563$) had survived (Staab *et al.*, 1981).

Because the preoperative CEA level is not linked prognostically to resectability or tumour extension, it provides additional prognostic information. However, a possible linkage between the preoperative CEA level and histological type or grading has not yet been evaluated. The corresponding investigations are proceeding.

The prognostic value of distinct ranges of preoperative CEA should facilitate the management of stomach-cancer patients for adjuvant postoperative treatment. A generalization from our results would have to be based on our methods. Other CEA test systems would be expected to involve different critical ranges of the preoperative CEA levels.

The authors thank Ms S. Glock for excellent technical assistance.

### REFERENCES

AMERICAN JOINT COMMITTEE FOR CANCER STAGING AND END RESULTS REPORTING (1977) *Manual for Staging of Cancer.* Chicago: American Joint Committee.

CONCANNON, J. P., DALBOW, M. H., HODGSON, S. E. & 5 others (1978) Prognostic value of preoperative carcinoembryonic antigen plasma levels in patients with bronchogenic carcinoma. *Cancer,* **42,** 1477.

EVANS, J. T., MITTELMAN, A., CHU, M., & HOLYOKE, E. D. (1978) Pre- and postoperative uses of CEA. *Cancer,* **42,** 1419.

FORD, C. H. J., STOKES, H. J. & NEWMAN, C. E. (1981) Carcinoembryonic antigen and prognosis after radical surgery for lung cancer: Immunochemical localization and serum levels. *Br. J. Cancer,* **44,** 145.

GOSLIN, R., STEELE, G., MCINTYRE, J. & 5 others (1980) The use of preoperative plasma CEA levels for the stratification of patients after curative resection of colorectal cancers. *Ann. Surg.,* **192,** 747.

INTERNATIONAL UNION AGAINST CANCER (1978) *TNM Classification of Malignant Tumours* 3rd Edn. (Ed. Harmer). New York: Springer-Verlag.

KJORSTAD, K. E. & ØRJASAETER, H. (1982) The prognostic value of CEA determinations in the plasma of patients with squamous cell cancer of the cervix. *Cancer* (in press).

KOHLER, J. P., SIMONOWITZ, D. & PALOYAN, D. (1980) Preoperative CEA level: A prognostic test in patients with colorectal carcinoma. *Am. Surg.*, **46**, 449.

PETO, R., PIKE, M. C., ARMITAGE, P. & 7 others (1976) Design and analysis of randomized clinical trials requiring prolonged observation of each patient. I. Introduction and design. *Br. J. Cancer*, **34**, 585. (1977) II. Analysis and examples *Br. J. Cancer*, **35**, 1.

PETO, R. & PETO, J. (1972) Asymptotically efficient rank invariant test procedures. *J. R. Statist. Soc. A*, **135**, 185.

STAAB, H. J., ANDERER, F. A., STUMPF, E. & FISCHER, R. (1980) CEA, ein nützlicher Indikator beim Magenkarzinom. In *Das Magenkarzinom, Frühdiagnose und Therapie* (Eds. Beger *et al.*). Stuttgart, G. Thieme-Verlag.

STAAB, H. J., ANDERER, F. A., BRÜMMENDORF, T., STUMPF, E., & FISCHER, R. (1981) Prognostic value of preoperative serum CEA level compared to clinical staging: I. Colorectal carcinoma. *Br. J. Cancer*, **44**, 652.

STOKES, T. C., STEVENS, J. F. S., LONG, P., LOCKEY, E. & MILLER, A. L. (1980) Preoperative carcinoembryonic antigen and survival after resection of lung cancer. *Br. J. Dis. Chest*, **74**, 390.

VINCENT, R. G., CHU, T. M. & LANE, W. W. (1979) The value of carcinoembryonic antigen in patients with carcinoma of the lung. *Cancer*, **44**, 685.

WANEBO, H. J., RAO, B., PINSKY, C. M. & 4 others (1978) Preoperative carcinoembryonic antigen level as a prognostic indicator in colorectal cancer. *N. Engl. J. Med.*, **299**, 448.